# Successful treatment with doxycycline monotherapy for human infection with *Babesia venatorum* (Babesiidae, Sporozoa) in China: a case report and proposal for a clinical regimen

**DOI:** 10.1186/s40249-023-01111-1

**Published:** 2023-07-13

**Authors:** Lei Huang, Yi Sun, Dan-Dan Huo, Ming Xu, Luo-Yuan Xia, Ning Yang, Wei Hong, Lin Huang, Wei-Min Nie, Ru-He Liao, Ming-Zhu Zhang, Dai-Yun Zhu, Yan Li, He-Cheng Ma, Xin Zhang, Yong-Gang Li, Xin-An Huang, Jing-Yuan Wang, Wu-Chun Cao, Fu-Sheng Wang, Jia-Fu Jiang

**Affiliations:** 1 Department of Infectious Diseases, The Fifth Medical Center of Chinese PLA General Hospital, Beijing, 100039 People’s Republic of China; 2 State Key Laboratory of Pathogen and Biosecurity, Beijing Institute of Microbiology and Epidemiology, Academy of Military Medical Sciences, Beijing, 100071 People’s Republic of China; 3 Inner Mongolia Medical University, Hohhot, 010059 People’s Republic of China; 4 School of Public Health, Shandong University, Jinan, 250100 People’s Republic of China; 5 The Center for Clinical Laboratory, The Fifth Medical Center of Chinese PLA General Hospital, Beijing, 100039 People’s Republic of China; 6 Artemisinin Research Center, Guangzhou University of Chinese Medicine, Guangzhou, 510000 People’s Republic of China

**Keywords:** Human babesiosis, *Babesia venatorum*, Doxycycline monotherapy, China

## Abstract

**Background:**

Human babesiosis is a worldwide disease caused by intraerythrocytic protozoa of the genus *Babesia*. It is transmitted by bites from ixodid ticks, and mechanically transmitted by blood transfusion. It is primarily treated with quinine and/or atovaquone, which are not readily available in China. In this study, we developed a novel treatment regimen involving doxycycline monotherapy in a patient with severe *Babesia venatorum* infection as an alternative therapeutic medication. The aim of our study is to provide a guidance for clinical practice treatment of human babesiosis.

**Case presentation:**

A 73-year-old man who had undergone splenectomy and blood transfusion 8 years prior, presented with an unexplained fever, headache, and thrombocytopenia, and was admitted to the Fifth Medical Center of the PLA General Hospital. He was diagnosed with *B. venatorum* infection by morphological review of thin peripheral blood smears, which was confirmed by multi-gene polymerase chain reaction (PCR), and sequencing of the entire 18s rRNA and partial β-tubulin encoding genes, as well as isolation by animal inoculation. The doxycycline monotherapy regimen (peros, 0.1 g bisindie) was administered following pharmacological guidance and an effective outcome was observed. The patient recovered rapidly following the doxycycline monotherapy. The protozoan load in peripheral blood samples decreased by 88% in hematocrit counts after 8 days, and negative PCR results were obtained after 90 days of follow-up at the hospital. The treatment lasted for 3 months without any side effects or sequelae. The nine-month follow-up survey of the patient did not reveal any signs of recrudescence or anti-babesial tolerance.

**Conclusions:**

We have reported a clinical case of successful doxycycline monotherapy for human babesiosis caused by *B. venatorum*, which provides an optional medical intervention for human babesiosis.

**Graphical Abstract:**

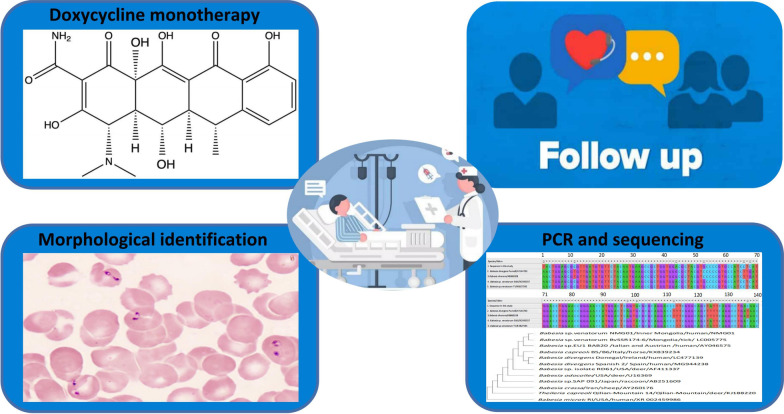

**Supplementary Information:**

The online version contains supplementary material available at 10.1186/s40249-023-01111-1.

## Background

Human babesiosis is a zoonotic disease caused by an intraerythrocytic protozoan of the genus *Babesia. Babesia* is a parasite seen in the red blood cells of mammals, and is transmitted mainly by tick bites and blood transfusions [1]. Currently, more than 100 species of *Babesia* are recognized as pathogenic to various wild and domestic animal hosts, including humans [2]. Since the first case of human babesiosis was reported in 1957 in a Croatian farmer (formerly Yugoslavia), who underwent splenectomy, the number of cases has increased dramatically, particularly in the Northern Hemisphere [3]. In the United States alone, the annual cumulative number of cases of human babesiosis was estimated to range from 20,000 to 24,000 between 2006 and 2018, and it has been designated as a notifiable infectious disease [4]. In China, several *Babesia* species have been recorded at least 317 human patients [5], including *Babesia divergens* [6], *B. venatorum* [7], *B. microti* [8], *B. crassa-*like pathogens [9], and other unassigned species [5].

*B. venatorum*, formerly known as *Babesia* sp. EU1, was named after the Austrian and Italian Babesia-infected splenectomized hunters [10]. Since then, protozoan species have been documented to naturally infect the host roe deer *Capreolus capreolus*, and the vector species *Ixodes ricinus* and *I. persulcatus* [11, 12]. In epidemiological surveys of Chinese patients, few human cases of infection with *B. venatorum* have been reported in the endemic regions of China [7, 13]. The overall disease burden of *B. venatorum* in China is typically underestimated, because of its mild manifestations and asymptomatic appearance. Consequently, human infections with *B. venatorum* tend to be ignored. An accurate diagnosis and effective treatment regimen for infection with *B. venatorum* is therefore urgently needed for clinical development and practice.

In this study, a patient with unexplained febrile symptoms sought medical services at the Fifth Medical Center of the PLA General Hospital. A morphological microscopic review of thin peripheral blood smear examination and multi-gene polymerase chain reaction (PCR) amplification testing, confirmed infection with *B. venatorum*, and appropriate treatment with sensitive doxycycline was administered.

Three generations of anti-*Babesia* drugs have been approved for clinical use. Quinine, a traditional drug used against *Babesia* and *Plasmodium* species, inhibits hemozoin biocrystallisation via the heme detoxification pathway, which facilitates the aggregation of cytotoxic heme and results in parasite death [2, 14, 15]. As a unique naphthoquinone with broad-spectrum anti-protozoal activity, atovaquone acts against susceptible parasites by inhibiting the mitochondrial electron transport chain at the site of the cytochrome bc1 complex (complex III), ultimately blocking the synthesis of nucleic acids and adenosine triphosphatase [16–18]. According to the clinical practice guidelines for diagnosis and management of human babesiosis, it is commonly recommended to treat human babesiosis with either quinine or atovaquone, in combination with clindamycin or azithromycin [2], since the two antibiotics are protein synthesis inhibitors of apicoplast, a plastid organelle in apicomplexan parasites [19–21]. Doxycycline, a broad-spectrum tetracycline-class antibiotic, is frequently used to treat *Plasmodium* spp. by killing erythrocytic-stage parasites that target the apicoplast [22], in combination with quinine. Several attempts have been made to demonstrate its distinctive anti-babesial effects in experimental animals [23–25]. However, to date, no trials with doxycycline have been conducted in human patients with *Babesia* infections.

## Case presentation

### Clinical course of the case

A 73-year-old male underwent splenectomy after a traffic accident in 2014, and blood transfusion was performed during surgery. He had no history suggestive of any chronic diseases, or drug or alcohol abuse. The patient, with no recorded family history, had quit smoking 10 years ago and lived with his family in an urban area of Hulunbuir grassland (E115°31’–126°04’, N 47°05’–53°20’) Inner Mongolia Autonomous Region, China.

**Fig. 1:**
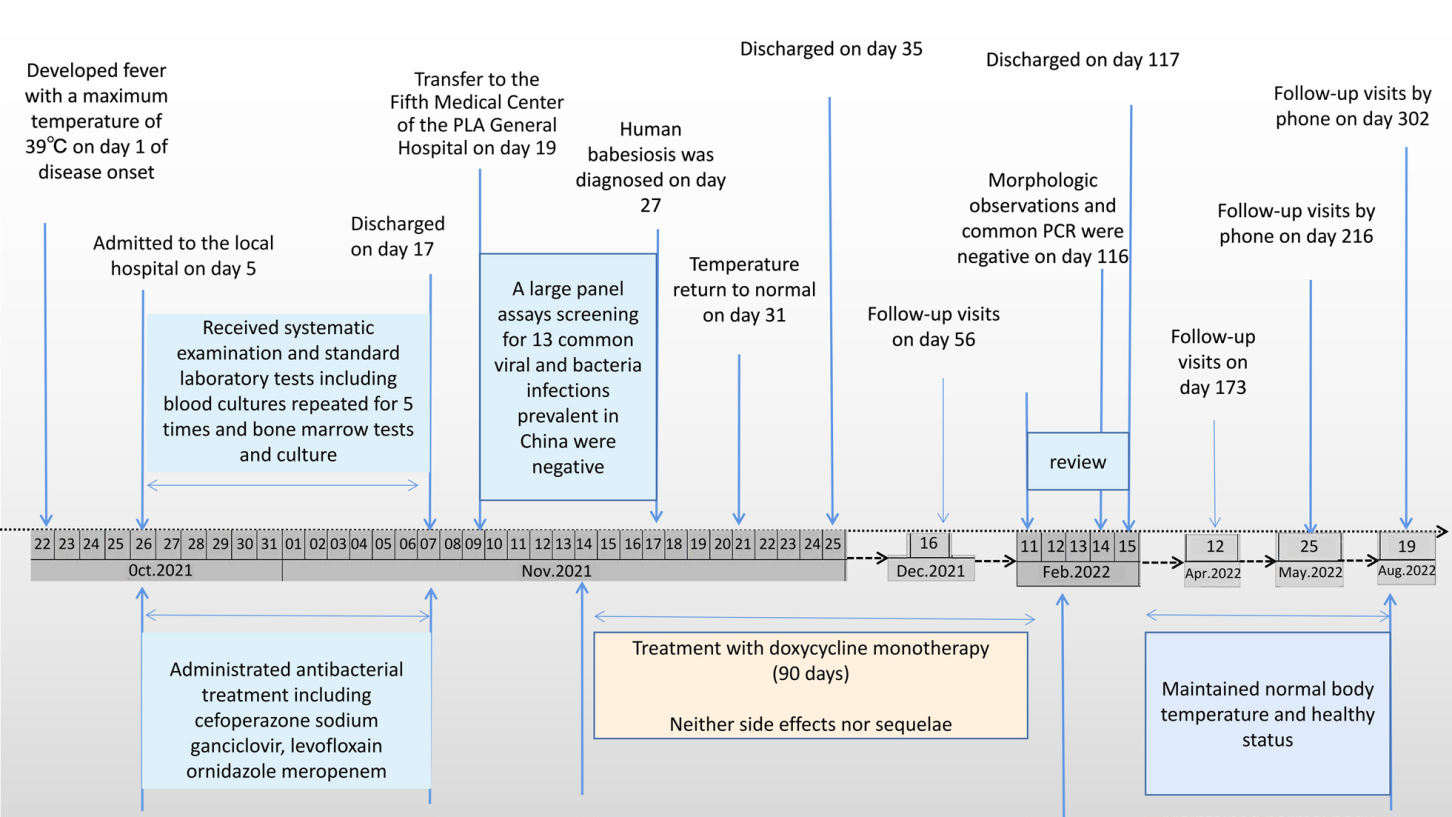
Timeline of doxycycline monotherapy for the patient infected with *Babesia venatorum* (Babesiidae, Sporozoa)

The patient developed fever, with a maximum body temperature of 39 °C on October 22, 2021, defined as the first day of the onset of the disease (Fig. 1). Headache, nausea, and vomiting persisted for several days. On October 26, the patient was admitted to the local hospital, where he underwent physical examination, standard laboratory tests, including blood cultures (five repeats) and bone marrow check and culture. He was administered antibacterial treatment, including cefoperazone sodium, ornidazole, and meropenem, over the following time (Additional file 1: Fig. S1). No relevant signs were found, except for a few abnormal biochemical tests, including thrombocytopenia (counts of platelets = 34 × 10^9^/L), increased C-reactive protein (CRP, 88.1 mg/L), and weakly positive tuberculosis-interferon gamma release assay. However, the patient’s condition did not improve.

**Fig. 2:**
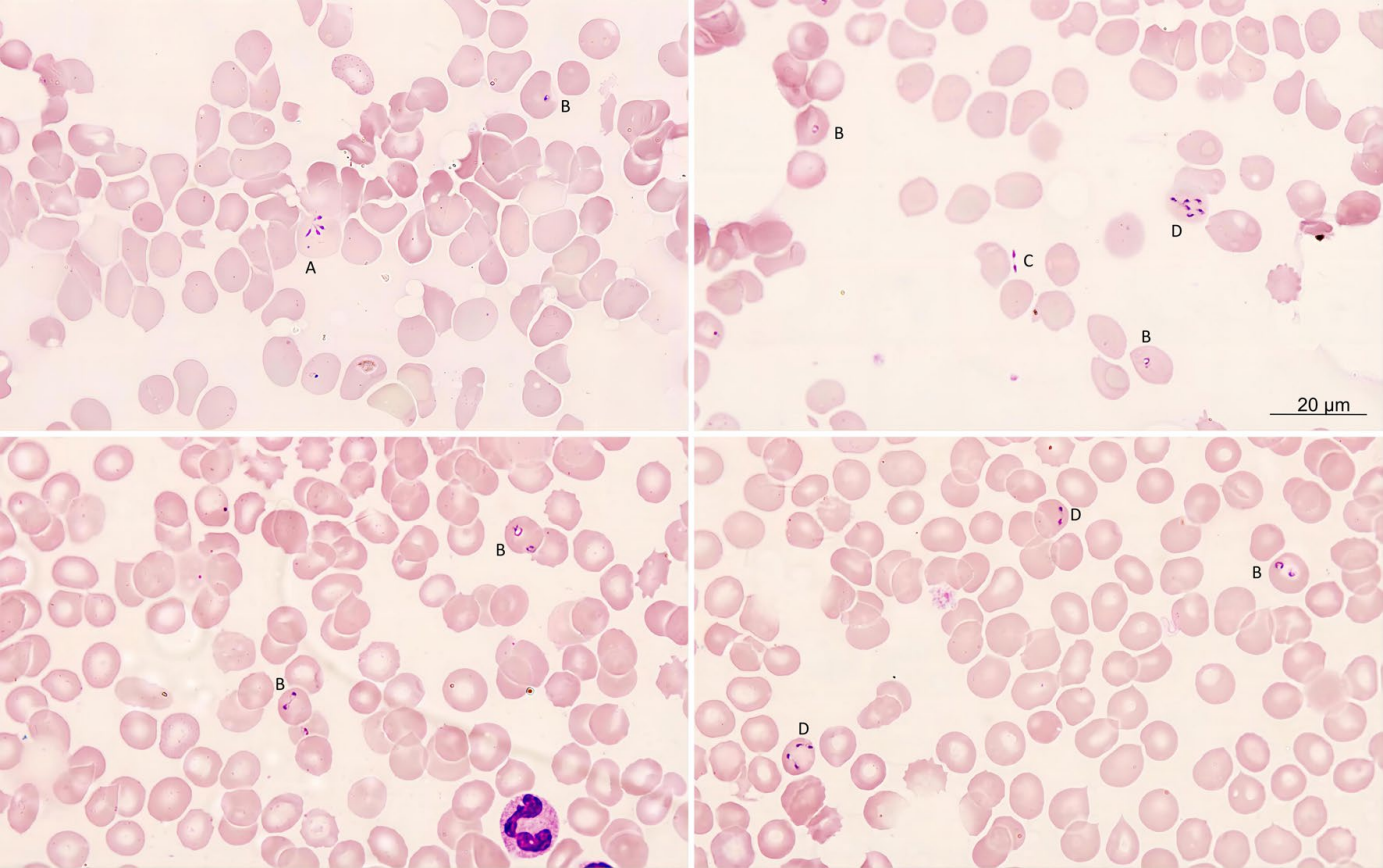
Photomicrographs of red cells infected with *Babesia venatorum* on a thin peripheral blood smear of the patient. **A** Tetrad; **B** Ring form trophozoites; **C** Extraerythrocytic *Babesia* parasites; **D** Schizont form

On November 9, 2021, the patient was transferred to the Fifth Medical Center of the PLA General Hospital in Beijing due to uncontrolled body temperature, with complain of no remission of fever, and dependence on indomethacin suppository twice daily. After admission, a detailed physical examination revealed no positive signs, except for abdominal scars owing to the previous splenectomy surgery. Assays for 11 pathogenic bacteria and two viruses (Additional file 1: Table S1) were performed at the hospital laboratory and no positive results were indicated. Therefore, we ruled out common bacterial and viral infections in this patient. Also, the erythrocyte sedimentation rate, pure protein derivative test, and computed tomography images of the lung did not indicate infection with tuberculosis. Notably, thrombocytopenia (counts of platelets = 31 × 10^9^/L) and extremely high ferritin levels (> 2000 ng/ml) were observed, and a tentative suspicion of hematological disorder or malignant tumor bone metastases was considered. To validate our suspicion, a thin smear of the peripheral blood sample was reviewed, on November 11, 2021. However, the clinical laboratory in the hospital did not report definitive positive results. We empirically used doxycycline monotherapy [peros (po), 0.1 g bisindie (Bid)] to treat some undetectable pathogens, as the patient’s symptoms deteriorated, and conventional the anti-infective therapy was insufficient. During the first two days of doxycycline administration, the patient’s body temperature gradually decreased, and the symptoms resolved. Hence, we submitted the blood sample drawn on November 11 to the Beijing Institute of Microbiology and Epidemiology for further pathogen detection. On November 17, a laboratory specialist reported that *Babesia* protozoan was found in the blood smears submitted on November 11 (Fig. 2). To confirm the result, we collected fresh blood samples on November 17 for smear observation, and submitted them for PCR testing and sequencing. *Babesia* was also found as expected in blood smears, and *B. venatorum* was identified with sequences annotation. Additionally, positron emission tomography-computed tomography on November 13, did not indicate any high metabolic lesions, and two aspirations for bone marrow on November 11 and 23, did not reveal any specific abnormalities. The decreased hemoglobin level was observed at day of 19, 20, 21, 32 and 35 (Additional file 1: Table S1), which suggest the anemia status of the patient. Meanwhile, the elevated lactate dehydrogenase demonstrated at day of 19, 20, 23 also indicated the hemolysis occurred in the acute phase, which consistent with the increased CRP level until the day 32 (Additional file 1: Table S2). Therefore, the patient was diagnosed with human babesiosis.

### *Babesia* species identification

**Fig. 3:**
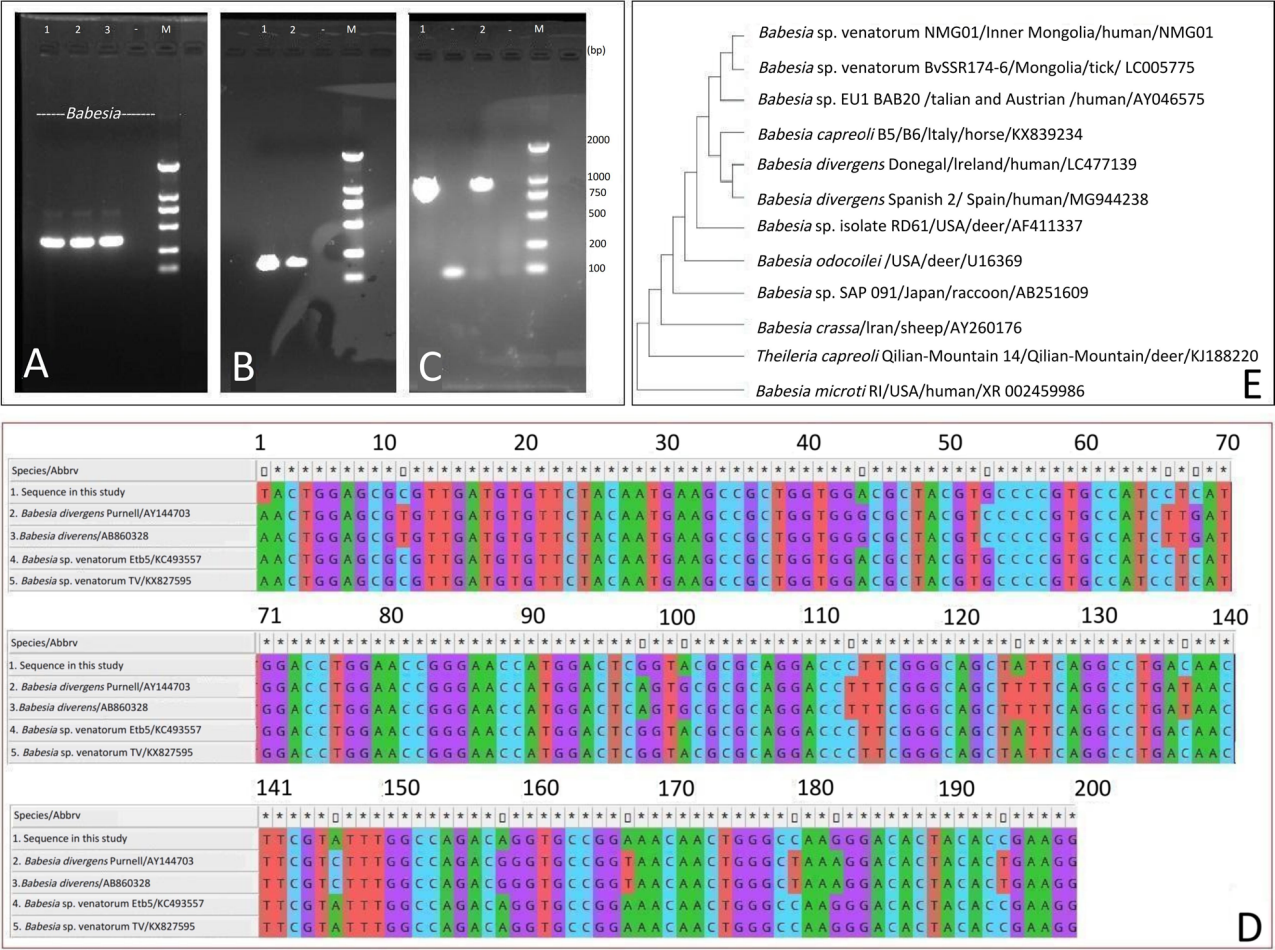
Molecular identification of the parasite in the patient from Inner Mongolia, China. **A** Electrophoresis of the partial18S rRNA target gene of *Babesia* spp. from human whole blood (Lanes 1−3); **B** Electrophoresis of the amplicon of a β-*tubulin* gene of *Babesia venatorum* from human whole blood (Lanes 1−2); **C** Electrophoresis of two amplicons of the entire 18S rRNA gene from *Babesia venatorum*; **D** Comparison analysis of the 199 base-pair nucleotide sequence alignment of babesia β-*tubulin* gene. **E** Phylogenetic analysis of the sequence from patient strain NMG01 and other members of the order Piroplasmide based on nearly entire 18S rRNA gene sequences, inferred using the use of the maximum likelihood method and bootstrap analysis of 1000 replicates to assess the reliability of the reconstructed phylogenies

Morphological examination, PCR testing, sequencing, parasitemia surveillance and isolation of inoculated experimental mice were performed to identify the babesia infection for the patient (Additional file 1). On microscopically reviewing the thin peripheral blood smears using an oil-immersion lens, typical intraerythrocytic ring-form trophozoites, tetrads, and paired pyriform (Fig. 2A–D) were discovered without any pigments and lesser quantities of merozoites, and the parasitemia level was calculated to be as high as 12,000 parasites/µl in a blood smear on November 11, 2021. The PCR results were positive only for *Babesia* spp. (Fig. 3A), but negative for other known tick-borne agents. The 373 bp sequence obtained indicated that the patient was infected with *B. venatorum*. A 199 bp *β-tubulin* gene amplicon also confirmed infection with *Babeisa* spp. (Fig. 3B), and its sequence (GenBank accession No. OP 522105) was identical to that of *B. venatorum* isolated from ticks (*Ixodes persulcatus*) (KX827595) (Fig. 3D). Two expected amplicons for the entire 18s rRNA gene (Fig. 3C) were also obtained, and its nearly full-length 18 S rRNA gene (1665 bp, OP559478) further confirmed the infection of *B. venatorum*, with 99.82% similarity to *B. venatorum* isolated from ticks (LC005775) in Mongolia and human patients in Europe (AY046575) within the same clade after phylogenetic analysis, the statistical software MEGA11 (Tamura, Stecher, and Kumar 2021,USA). (Fig. 3E). Red blood cells infected with *Babesia* were also seen in the blood smears of the inoculated severe combined immune deficiency (SCID) mice on days 3, 6, and 12. Finally, human babesiosis was diagnosed according to the recommended laboratory criteria specified by the Centers for Disease Control and Prevention (CDC) [2].

### Treatment, outcomes and follow up

**Fig. 4:**
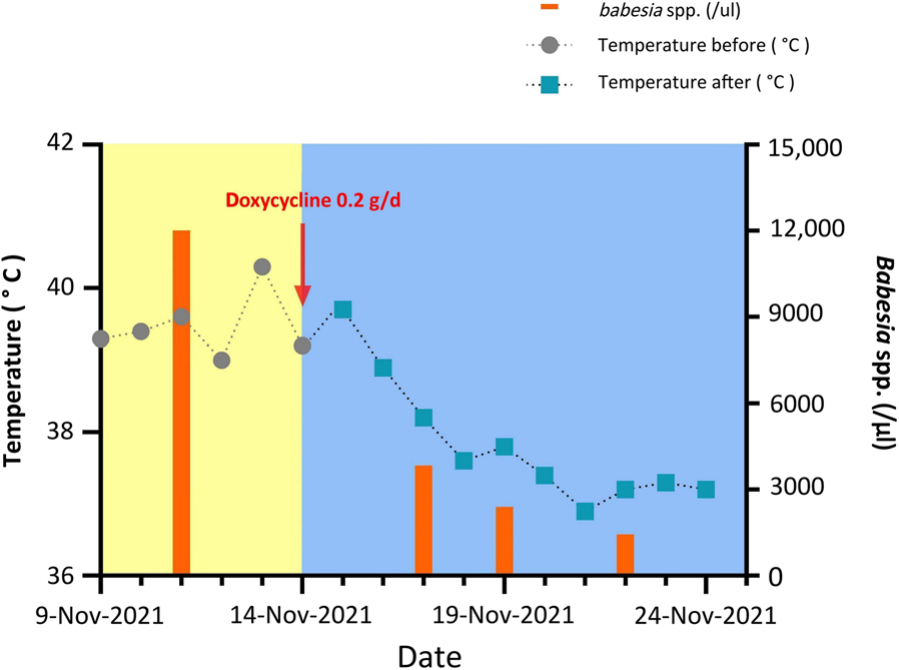
Dynamic change in the patient’s temperature and *Babesia* spp. infected erythrocyte count following the administration of doxycycline monotherapy

After the patient was diagnosed with human babesiosis, following the expert opinion on anti-babesial theory on European human babesiosis specified in 2020 [19], we were advised to treat the patient with atovaquone or quinine, accompanied by either clindamycin or azithromycin, since *B. venatorum* is the closest relatives of *B. divergens* in *Babesia* sensu stricto lineage. However, an emergency prescription for either quinine or atovaquone is inaccessible in Beijing. As an alternative chemical, doxycycline has been shown to have sensitive antiparasitic activities by inhibiting the synthesis of apicoplast protein in *Plasmodium* species, and successfully treating *B. canis* and *B. gibsoni* infection in dogs [23, 24]. It is reported that doxycycline was also aimed at other possible pathogens [26]. Notably, the patient’s clinical symptoms gradually improved following the administration of doxycycline. Therefore, we continued our monotherapy of doxycycline (po, 0.1 g Bid), followed by strict supervision and regular monitoring of parasite loads. As expected, the patient recovered rapidly, and the *Babesia* protozoan burden decreased dramatically from the pretreated 12,000/µl to 3840/µl [3 days post days post treatment (dpt)], 2400/µl (5 dpt), and 1440/µl (8 dpt) (Fig. 4). Broken or fragmented merozoites were frequently recorded during the regimen. Body temperature decreased after the two-day therapy, returned to normal after seven-day therapy, and then maintained normally thereafter (Fig. 4). The clinical manifestations subsided (Additional file 1: Table S3).

The patient was discharged on the 35th day after the onset of the disease. Since then, a consecutive follow-up survey of the patient with a continuing course of doxycycline treatment was performed for 90 days. The patient was readmitted to the hospital for follow-up on February 11, 2022. We performed a detailed physical examination and found no abnormalities. The same laboratory protocol was used to monitor the *Babesia* burden in patients. Morphological examination and PCR tests were negative for whole blood samples collected on February 15, 2022, 117 days after the onset time (Fig. 1). No doxycycline-related side effects or sequelae, including erythema or hepatic insufficiency, were observed. The patient maintained a normal body temperature and health status from the time the chemical therapy was stopped to the final follow-up visit on August 19, 2022 (Fig. 1 and Additional file 1: Table S3).

## Discussion and conclusion

We confirm a case of human babesiosis in Inner Mongolia, China according to the guidelines on diagnosis and management of babesiosis [2]. The first obtained entire 18s rRNA and β-tubulin protein genes were from a patient infected with *B. venatorum* in China. To our knowledge, this is the first successful clinical application of doxycycline monotherapy for human babesiosis, providing an alternative therapeutic strategy with clinical trials and useful anti-babesial chemicals.

Cycline reportedly has satisfactory prophylactic effects against experimental infections with highly pathogenic strains of *B. canis* and *B. bovis* [23–25]. However, it is not recommended as a first-line treatment for *Babesia* infection, even when the parasite is doxycycline-sensitive, because the anti-parasite effect of doxycycline is delayed [27]. In the present case of human babesiosis, the patient maintained a normal body temperature and health status from the time that the therapy was stopped, until the final follow-up visit. This suggests that doxycycline is suitable for the treatment of babesiosis in humans.

An epidemiological survey of possible infection routes did not produce definitive results, similar to the previous cases of human babesiosis. *Babesia* spp. were most frequently transmitted by *Ixodes* spp. ticks such as *B. microti* detected in *Dermacentor nuttallii*, and *Hyalomma asiaticum* from the Ceke, Mandala, and Manzhouli areas of Inner Mongolia, China [28, 29]. *Babesia* infections may also occur as a result of blood transfusions [30]. Although no definitive tick bites were recorded in our patient, humans are often imperceptible to tick bites, and hence these cannot be excluded. Another possible mode of infection, caused by a previous blood transfusion surgery, should not be discounted. Cases of human babesiosis relevant to blood transfusion have been documented for *B. microti* since 1980 [31], and for *B. duncani* since 1994 [32]. The incubation period of these *Babesia* species ranges from 23 to 384 days, when transmitted by blood transfusion [33]. As for *B. venatorum*, no human babesiosis cases were recorded to be caused by blood transfusions or other relevant operations. One splenectomy patient from Germany without obvious exposure history for tick bite and blood product developed chronic *B. venatorum* (formerly named *Babesia* sp. EU1) parasitemia after treatment with quinine and clindamycin due to a relapse of Hodgkin’s disease, and long-term maintenance therapy with atovaquone was administered to clear the parasites [34]. Our case of human babesiosis remained undiagnosed for eight years after splenectomy, and blood transfusions had to be given. Consequently, *B. venatorum* exceeded the limit of incubation period in this patient, and the clinical manifestations might have been caused by a possible recrudescence, or an unrecorded tick bite, especially in the asymptomatic period.

Notably, monotherapy for human babesiosis has been successful. It is reasonable to attribute the inhibitory activity of doxycycline on protein synthesis in the apicoplast of the parasites. As both *Babesia* and *Plasmodium* parasites replicate inside the erythrocytes of the mammalian host, the apicoplast, a special organelle required to invade host erythrocytes, may serve as a candidate target for anti-parasite chemicals [30]. To inhibit protein synthesis in apicoplasts, future trials should address potential anti-babesial chemicals, such as tetracyclines (tetracycline, doxycycline, and minocycline), macrolides (spiramycin and azithromycin), and lincosamides (lincomycin, clindamycin, pirlimycin, and chloramphenicol) [35]. In 1996, the prophylactic treatment of experimental canine babesiosis (*B. canis*) with doxycycline achieved success, with satisfactory efficacy [23]. Another option is an alternative combination therapy regimen, which works well with metronidazole, clindamycin, and doxycycline for *B. gibsoni* (Asian genotype) infection in dogs in Hong Kong [24]. In China, clindamycin is used to treat patients with *B. microti* infections. Azithromycin and atovaquone was used for treatment of one pediatric patient infected with *B. venatorum* in 2014 [13], and three cases of *B. microti* [36–38]. Doxycycline, a tetracycline antibiotic, is widely used to treat various pathogens [26, 39, 40]. Currently, there is no standard course of treatment owing to the lack of medical records on doxycycline monotherapy for human *Babesia* infections. In the present study, we used doxycycline monotherapy for 90 days to prevent recrudescence, and found no adverse drug reactions, as revealed by the patient’s health status and laboratory evidence. This implies that doxycycline monotherapy for human infection with *B. venatorum* is suitable for up to 90 days.

In China, the actual number of infected individuals, including blood product donors may be significantly higher than expected, as many of them may be asymptomatic. However, most doctors and laboratory personnel in China lack awareness about human babesiosis, leading to underdiagnosis and an improper treatment. Similar to this case, laboratory personnel are also prone to miss the protozoan, as they are accustomed to fetching results directly from the hematology analyzer. Furthermore, due to insufficient awareness of the disease, first-line drugs for human babesiosis are still scarce in China. Therefore, doxycycline monotherapy should be considered in the future.

In summary, our successful experience treatment of human babesiosis caused by *B. venatorum* strongly suggests that doxycycline could be used as an alternative chemical to treat natural *Babesia* protozoal infections. However, it is necessary to expand the number of cases and perform more standard case-control studies to further study the efficacy and duration of doxycycline monotherapy.

## Supplementary Information

**Additional file 1. Figure S1.** The administration time and the dosage daily of the drugs before the patient was transferred to the Sentinel Hospital of the FifthMedical Center of the PLA General Hospital. **Table S1.** The panel assaysscreening for 13 common viruses and bacteria. **Table S2.** Laboratory values observed during hospitalisation (Reference values are given in parentheses). **Table S3.** Symptoms and signs reported during thefirst hospital stay. Additional materials.

## Data Availability

All data generated or analyzed relating to this study are presented within this published article.
