# Correction: Successful treatment with doxycycline monotherapy for human infection with *Babesia venatorum* (Babesiidae, Sporozoa) in China: a case report and proposal for a clinical regimen

**DOI:** 10.1186/s40249-023-01123-x

**Published:** 2023-07-27

**Authors:** Lei Huang, Yi Sun, Dan‑Dan Huo, Ming Xu, Luo‑Yuan Xia, Ning Yang, Wei Hong, Lin Huang, Wei‑Min Nie, Ru‑He Liao, Ming‑Zhu Zhang, Dai‑Yun Zhu, Yan Li, He‑Cheng Ma, Xin Zhang, Yong‑Gang Li, Xin‑An Huang, Jing‑Yuan Wang, Wu‑Chun Cao, Fu‑Sheng Wang, Jia‑Fu Jiang

**Affiliations:** 1 Department of Infectious Diseases, The Fifth Medical Center of Chinese PLA General Hospital, Beijing, 100039 People’s Republic of China; 2 State Key Laboratory of Pathogen and Biosecurity, Beijing Institute of Microbiology and Epidemiology, Academy of Military Medical Sciences, Beijing, 100071 People’s Republic of China; 3 Inner Mongolia Medical University, Hohhot, 010059 People’s Republic of China; 4 School of Public Health, Shandong University, Jinan, 250100 People’s Republic of China; 5 The Center for Clinical Laboratory, The Fifth Medical Center of Chinese, PLA General Hospital, Beijing, 100039 People’s Republic of China; 6 Artemisinin Research Center, Guangzhou University of Chinese Medicine, Guangzhou, 510000 People’s Republic of China

**Correction: Infect Dis Poverty (2023) 12: 67** **https://doi.org/10.1186/s40249-023-01111-1**

Following publication of the original article [1], the authors reported that the affiliations to authors Ming-Zhu Zhang and Jing-Yuan Wang were mistakenly assigned.

The correct affiliations of Ming-Zhu Zhang should be:

^2^State Key Laboratory of Pathogen and Biosecurity, Beijing Institute of Microbiology and Epidemiology, Academy of Military Medical Sciences, Beijing, 100071, People’s Republic of China

^4^School of Public Health, Shandong University, Jinan, 250100, People’s Republic of China

The correct affiliation of Jing-Yuan Wang should be:

^3^Inner Mongolia Medical University, Hohhot, 010059, People’s Republic of China

The original article [1] has been updated.

## References

[1] Huang L, Sun Y, Huo DD (2023). Successful treatment with doxycycline monotherapy for human infection with *Babesia venatorum* (Babesiidae, Sporozoa) in China: a case report and proposal for a clinical regimen. Infect Dis Poverty.

