# Supplementary material for: Successful treatment with doxycycline monotherapy for human infection with Babesia venatorum (Babesiidae, Sporozoa) in China: a case report and proposal for a clinical regimen
Source: Infect Dis Poverty. 2023 Jul 13;12:67. doi: 10.1186/s40249-023-01111-1 (PMC10339522; doi:10.1186/s40249-023-01111-1)
Supplement: Supplementary file 1 — Additional file 1. Figure S1. The administration time and the dosage daily of the drugs before the patient was transferred to the Sentinel Hospital of the FifthMedical Center of the PLA General Hospital. Table S1. The panel assaysscreening for 13 common viruses and bacteria. Table S2. Laboratory values observed during hospitalisation (Reference values are given in parentheses). Table S3. Symptoms and signs reported during thefirst hospital stay. Additional materials. [file 40249_2023_1111_MOESM1_ESM.docx]

**Additional file 1**


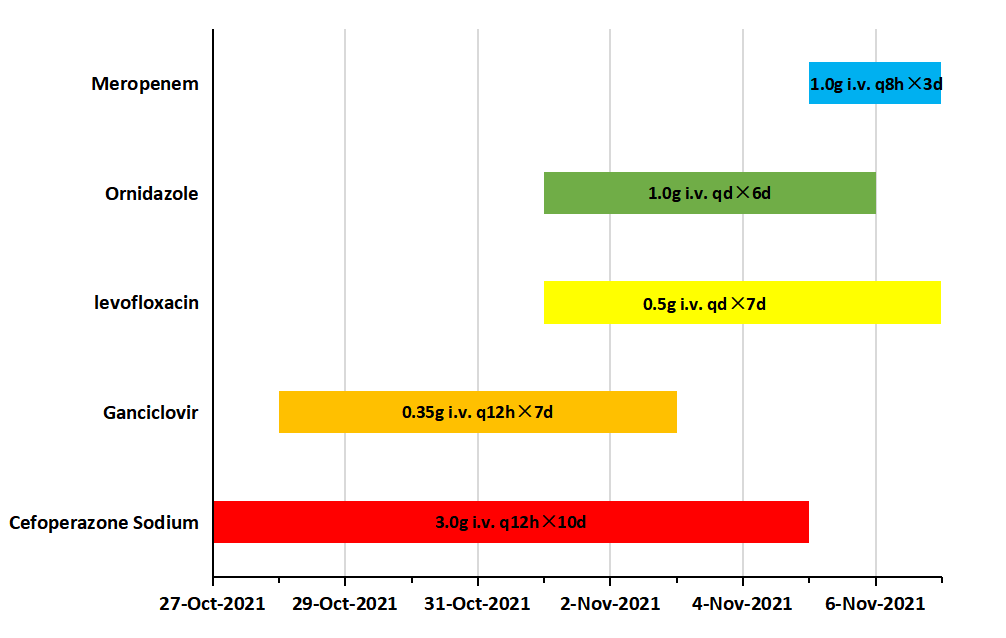
 **Additional file 1: Figure S1. The administration time and the dosage daily of the drugs before the patient was transferred to the Sentinel Hospital of the Fifth Medical Center of the PLA General Hospital.**

The exact drug dosage is Cefoperazone Sodium 3 g, q12h, Ganciclovir 0.35 g, q12h, Levofloxacin 0.5 g, qd, Ornidazole 0.5 g, q12h, Meropenem 1 g, qd.

**Additional file 1: Table S1. The panel assays screening for 13 common viruses and bacteria ^*^**

| **Pathogens** | **Testing Kits** | **Method** |
| --- | --- | --- |
| Epidemic Typhus | The Wei-Felix test  （Typhus paratyphus and proteobacteria OX19, OX2, OXK diagnostic bacterial fluid, 2173404340） | Agglutination test  (test tube method) |
| Endemic Typhus |  |  |
| North Asian tick-borne rickettsiosis |  |  |
| Mediterranean exanthematous fever |  |  |
| Rickettsia australis |  |  |
| Kew Gardens spotted fever |  |  |
| Akamushi disease |  |  |
| Brucellic disease | The Brucella agglutination assay  （The Brucella Antibody Detection Kit，2073403127） | Agglutination test  (test tube method) |
| Typhoid fever | The Widal's reaction  （Typhus paratyphus and proteobacteria OX19, OX2, OXK diagnostic bacterial fluid, 2173404340） | Agglutination test  (test tube method) |
| Paratyphoid fever |  |  |
| EB virus infections（EBVDNA） | （EB Virus Nucleal Acid Detection Kit, 2017340077） | The PCR-fluorescence probe method |
| CMV virus infections  （CMVDNA） | （The Human Cytomegalovirus Nucleic Acid Detection Kit, 20173400080） | The PCR-fluorescence probe method |
| Lyme disease | The Lyme disease BB antibody protein blot assay  (IgG detection kit for anti-Spirospira antibody, Scientific research reagent, DN 2131-3201 G LS) | immunoblotting |

*：The patient was diagnosed fever of unknown origin (FUO) at first, and aetiology testing was performed immediately after being admitted to our hospital. The Wei-Felix test (Typhus paratyphus and proteobacteria OX19, OX2, OXK diagnostic bacterial fluid，2173404340) was negative, Epidemic Typhus, Endemic Typhus, North Asian tick-borne rickettsiosis, Mediterranean exanthematous fever, *Rickettsia australis*, Kew Gardens spotted fever, and akamushi disease were excluded. The Brucella agglutination assay（The Brucella Antibody Detection Kit，2073403127）was negative, Brucellic disease was ruled out. The Widal's reaction shows Eberthella typhi H and O were negative, *Salmonella paratyphi* C was 1:160, but the patient was not have coeliodynia and alvi profluvium, Typhoid fever and Paratyphoid fever were ruled out. The EBV DNA （Epstein-Barr Virus Nucleal Acid Detection Kit, 2017340077）and CMV DNA（The Human Cytomegalovirus Nucleic Acid Detection Kit, 20173400080）were both negative，EBV and CMV infection was also ruled out. The Lyme disease BB antibody protein blot assay (IgG detection kit for anti-Spirospira antibody, Scientific research reagent, DN 2131-3201 G LS) shows weakly positive for surface protein C p25 and flagellin p41++, but the patient did not have erythema nodosum migrans and arthrophlogosis during disease process. There was insufficient evidence for the diagnosis of Lyme disease.

**Additional file 1: Table S2. Laboratory values observed during hospitalisation （Reference values are given in parentheses）**

| TIME | 2021-11-9  (Day19) | 2021-11-10  (Day20) | 2021-11-11  (Day21) | 2021-11-12  (Day22) | 2021-11-13  (Day23) | 2021-11-17  (Day27) | 2021-11-22  (Day32) | 2021-11-25  (Day35) | 2021-12-16  (Day56) | 2022-4-12  (Day173) |
| --- | --- | --- | --- | --- | --- | --- | --- | --- | --- | --- |
| RBC (4.09–5.74 × 10^12^/L) | 4.23 | 4.04 | 3.82 | N/A | 4.28 | N/A | 3.21 | 3.53 | N/A | 4.56 |
| Hb  (131–172 g/L) | 137 | 126 | 120 | N/A | 135 | N/A | 101 | 112 | N/A | 145 |
| Hct  (38.0–50.8%) | 40.50 | 37 | 36.3 | N/A | 40.9 | N/A | 30.8 | 34 | N/A | 43.20 |
| MCV  (83.9–99.1 fL) | 95.7 | 91.6 | 94.9 | N/A | 95.5 | N/A | 96 | 96.3 | N/A | 94 |
| MHC (27.8–33.8 pg) | 32.5 | 31.2 | 31.3 | N/A | 31.5 | N/A | 31.5 | 31.7 | N/A | 31.8 |
| MCHC (320–355 g/L) | 339 | 341 | 330 | N/A | 330 | N/A | 328 | 329 | N/A | 336 |
| WBC (3.97–9.15 × 10^9^/L) | 5.89 | 6.74 | 11.83 | N/A | 13.24 | 8.30 | 10.51 | 767 | N/A | 6.32 |
| NEUT  (2–7 × 10^9^/L) | 2.64 | 2.84 | 3.52 | N/A | 4.71 | 2.1 | 2.53 | 2.56 | N/A | 2.84 |
| LYM (0.8–4.0 × 10^9^/L) | 2.18 | 3.07 | 6.7 | N/A | 7.05 | 4.67 | 6.15 | 4.06 | N/A | 2.83 |
| BASO  (0–11 × g/L) | 0.01 | 0.02 | 0.06 | N/A | 0.05 | 0.02 | 0.03 | 0.01 | N/A | 0.00 |
| MONO (0.12–1.0 × 10^9^/L) | 1.03 | 0.81 | 1.55 | N/A | 1.42 | 1.45 | 1.78 | 4.06 | N/A | 0.56 |
| EO  (0.02–0.5 × 10^9^ /L) | 0.00 | 0.00 | 0.00 | N/A | 0.01 | 0.06 | 0.02 | 0.02 | N/A | 0.09 |
| PLT  (85–303 × 10^9^ /L) | 41 | 31 | 36 | N/A | 52 | 76 | 128 | 130 | N/A | 179 |
| aPTT  (23–42 sec) | N/A | 45.3 | N/A | N/A | N/A | 40.5 | N/A | N/A | N/A | N/A |
| INR  (0.8–1.2) | N/A | 1.16 | N/A | N/A | N/A | 1.13 | N/A | N/A | N/A | N/A |
| PT  (10.2–14.3 sec) | N/A | 13.1 | N/A | N/A | N/A | 12.8 | N/A | N/A | N/A | N/A |
| Glc (3.9–6.1 mmol/L) | 6.3 | 5.1 | N/A | N/A | N/A | 5.6 | N/A | N/A | N/A | N/A |
| Na (136–145 mmol/L) | 133 | 131 | N/A | N/A | N/A | 131 | 133 | N/A | N/A | N/A |
| K  (3.5–5.2 mmol/L) | 3.6 | 3.9 | N/A | N/A | N/A | 3.6 | 4.8 | N/A | N/A | N/A |
| Cl  (93–108 mmol/L) | 99.8 | 99 | N/A | N/A | N/A | 98 | 101.5 | N/A | N/A | N/A |
| tCO2 (23–29 mmol/L) | 26 | 25 | N/A | N/A | N/A | 29 | 28 | N/A | N/A | N/A |
| Mg (0.7–1.1 mmol/L) | N/A | 0.8 | N/A | N/A | N/A | N/A | N/A | N/A | N/A | N/A |
| Ca (2.08–2.6 mmol/L) | N/A | 1.85 | N/A | N/A | N/A | N/A | N/A | N/A | N/A | N/A |
| BUN (2.9–8.2 mmol/L) | 7.08 | 7.1 | N/A | N/A | N/A | 5.0 | 4.9 | N/A | N/A | 7.2 |
| CR (62–115 µmol/L) | 73 | 67 | N/A | N/A | N/A | 71 | 72 | N/A | N/A | N/A |
| CRP (0.068–8.2 mg/L) | 49.14 | 63.8 | N/A | 54.95 | 51.44 | N/A | 13.9 | N/A | N/A | 62 |
| PCT  （0–0.5 ng/ml） | 0.499 | 0.671 | N/A | 9.82 | 4.81 | 1.78 | 0.302 | N/A | N/A | N/A |
| ALB  (35–55 g/L) | 27 | 25 | N/A | N/A | N/A | N/A | 28 | N/A | N/A | N/A |
| AST  (8–40 U/L) | 36 | 39 | N/A | N/A | N/A | N/A | 65 | N/A | 40 | 23 |
| ALT  (5–40 U/L) | 21 | 18 | N/A | N/A | N/A | N/A | 44 | N/A | 41 | 18 |
| CK  (38–174 U/L) | 39 | 37 | N/A | N/A | N/A | N/A | N/A | N/A | N/A | N/A |
| GGT  (11–50 U/L) | 75 | 67 | N/A | N/A | N/A | N/A | 48 | N/A | 78 | N/A |
| AP  (40–150 U/L) | 79 | 70 | N/A | N/A | N/A | N/A | 70 | N/A | N/A | N/A |
| LDH  (U/L) | 487 | 560 | N/A | N/A | N/A | N/A | 526 | N/A | N/A | N/A |
| tBILI (3.4–20.5 µmol/L) | 26.7 | 28.2 | N/A | N/A | N/A | N/A | 19.3 | N/A | 11.5 | 12.3 |
| tAMY (28–100 U/L) | 42 | 34 | N/A | N/A | N/A | N/A | N/A | N/A | N/A | N/A |
| t PR  (60–83 g/L) | 63 | 57 | N/A | N/A | N/A | N/A | N/A | N/A | N/A | N/A |
| CHOL (2.8–5.2 mmol/L) | N/A | 1.4 | N/A | N/A | N/A | N/A | N/A | N/A | N/A | 4.04 |
| HDL(1.16–1.42 mmol/L) | N/A | 0.3 | N/A | N/A | N/A | N/A | N/A | N/A | N/A | 1.22 |
| TG(0.56–1.7 mmol/L) | N/A | 1.41 | N/A | N/A | N/A | N/A | N/A | N/A | N/A | 0.87 |
| LDL  (2.1–3.1 mmol/L) | N/A | 0.8 | N/A | N/A | N/A | N/A | N/A | N/A | N/A | 2.94 |

RBC = red blood cell count; Hb = haemoglobin; Hct = haematocrit; MCV = mean corpuscular volume; MHC = mean corpuscular haemoglobin; MCHC = mean corpuscular haemoglobin concentratio; WBC = white blood cell count; LYM = lymphocytes; BASO=basophiles, MONO=monocytes; EO=eosinophiles; PLT = platelets; aPTT = activated partial thromboplasmin time; INR = international normalized ratio; PT = prothrombin time; Glc = glucose; Na = sodium; K = potassium; Cl = chloride; tCO2 = total carbon dioxide; Mg = magnesium; Ca = ionized calcium; BUN = blood urea nitrogen; CR = creatinine; CRP = C-reactive protein; ALB = albumin; AST = aspartate aminotransfrease; ALT = alanine aminotransferase; CK = creatine kinase; GGT = gamma-glutamyl transferase; AP = alkaline phosphatase; LDH = lactate dehydrogenase; tBILI = total bilirubin; tAMY = total amylase; tPR = total protein; LAC = lactate; CHOL = cholesterol; HDL = high-density lipoprotein; TG = triglyceride; LDL = low-density lipoprotein;

N/A=No Date.

**Additional file 1: Table S3: Symptoms and signs reported during the first hospital stay**

| **Time** | **Temperature (°C)** | **Arterial pulse**  **（time / minute）** | **Blood pressure （mmHg）** | **Respire**  **（time / minute）** | **Headache** | **Fatigue** | **Oxygen need** | **Dizziness** | **Vomit** |
| --- | --- | --- | --- | --- | --- | --- | --- | --- | --- |
| **2021-11-9**  **(Day 19)** | **39.3** | **107** | **128/89** | **20** | **+** | **+** | **2L** | **-** | **-** |
| **2021-11-10**  **(Day 20)** | **39.4** | **115** | **128/81** | **22** | **+** | **+** | **2L** | **-** | **-** |
| **2021-11-11**  **(Day 21)** | **39.6** | **116** | **137/76** | **25** | **+** | **+** | **2L** | **-** | **-** |
| **2021-11-12**  **(Day 22)** | **39.1** | **110** | **121/80** | **24** | **-** | **+** | **2L** | **-** | **-** |
| **2021-11-13**  **(Day 23)** | **40.3** | **110** | **125/85** | **20** | **-** | **+** | **2L** | **-** | **-** |
| **2021-11-14**  **(Day 24)** | **39.2** | **106** | **111/74** | **20** | **-** | **-** | **2L** | **-** | **-** |
| **2021-11-15**  **(Day 25)** | **38.7** | **108** | **125/75** | **21** | **-** | **-** | **2L** | **+** | **-** |
| **2021-11-16**  **(Day 26)** | **38.9** | **108** | **130/85** | **21** | **-** | **-** | **2L** | **-** | **20ml** |
| **2021-11-17**  **(Day 27)** | **38.2** | **102** | **124/77** | **20** | **-** | **-** | **2L** | **-** | **-** |
| **2021-11-18**  **(Day 28)** | **37.6** | **96** | **125/73** | **18** | **-** | **-** | **NO** | **-** | **-** |
| **2021-11-19**  **(Day 29)** | **37.8** | **96** | **132/88** | **20** | **-** | **-** | **NO** | **-** | **-** |
| **2021-11-20**  **(Day 30)** | **37.4** | **90** | **123/76** | **18** | **-** | **-** | **NO** | **-** | **-** |
| **2021-11-21**  **(Day 31)** | **36.9** | **92** | **127/76** | **19** | **-** | **-** | **NO** | **-** | **-** |
| **2021-11-22**  **(Day 32)** | **37.2** | **98** | **128/87** | **18** | **-** | **-** | **NO** | **-** | **-** |
| **2021-11-23**  **(Day 33)** | **37.3** | **93** | **135/83** | **18** | **-** | **-** | **NO** | **-** | **-** |
| **2021-11-24**  **(Day 34)** | **37.2** | **92** | **119/79** | **18** | **-** | **-** | **NO** | **-** | **-** |
| **2021-11-25**  **(Day 35)** | **37.1** | **95** | **122/80** | **20** | **-** | **-** | **NO** | **-** | **-** |

Temperature, Arterial pulse, Blood pressure, Respire values indicated are the highest measured for each day. The clinical symptoms of the early-stage patients include, headache, fatigue, dizziness and vomit. Oxygen was delivered by nasal cannula; values are expressed in litres/min. + present; - absent.

**Methods for Babesia species identification**

**Morphological examination**

A thin smear of the peripheral blood sample was prepared according to the conventional method, fixed with methanol, stained with 1% Giemsa solution for 30 min, and observed under a microscope using an oil-immersion lens. The numbers of protozoa and red blood cells were counted in 100 positive fields. The density of the protozoa was calculated as the number of protozoa per microliter of blood.

**PCR testing, sequencing and analysis**

Whole blood was screened for *Babesia* and other tick-borne pathogens with primers for the *Babesia*-specific 18S rRNA gene ^[1]^, *Anaplasma*-specific partial 16S rRNA gene ^[2]^, *Rickettsia*-specific *ompA* gene ^[3]^, *Borrelia miyamotoi*-specific 16S rRNA gene ^[4]^, and *Borrelia burgdorferi*-specific 5S-23S rRNA gene ^[5]^, respectively. Target amplicons were submitted to Beijing Tianyi Huiyuan Biotechnology Co. LTD for bidirectional sequencing. The obtained sequences were compared using the online BLAST software in NCBI (https://blast.ncbi.nlm.nih.gov/Blast.cgi). The *β-tubulin* gene of *B. venatorum* was also amplified using the primers BabtubF 5'- ACTGGAGCGCGTTGATGTGTTCT -3' and BabtubR 5'- GCCTTCGGTGTAGTG TCCCTTGG -3', designed according to the reference gene (KX827595). Meanwhile, the full-length 18S rRNA gene of *B. venatorum* was amplified with semi-nested PCR by the out primers babe16s-1F (5'- GCCA GTAGTCATATGCTTGTCTTAA -3') and babe16s-1666R (5'- CTCCTTC CTTTAAGTGATAAGGTTC-3'), and the inner primers babe16s-916R (5'- GGTATCTGATCGTCTTCGATCCCCT -3') and babe16s-730F (5'- TTTGGTTCTATTTTGTT GGTTTTTG -3'), respectively, designed according to the reference genes LC005775 and KU204792. The sequences of the two amplicons obtained from the second PCR were spliced using the CLC Genomics Workbench software (QIAGEN CLC Bio, Dusseldorf, Germany).

**Laboratory animal inoculation**

The patient’s blood was injected into six severe combined immunodeficiency (SCID) mice via the caudal vein and intraperitoneal cavity. Tail blood was collected every three days for blood smears to monitor parasitemia over a period of 27 days.

**References**

1. Jiang JF, Zheng YC, Jiang RR, Li H, Huo QB, Jiang BG, et al. Epidemiological, clinical, and laboratory characteristics of 48 cases of "*Babesia venatorum*" infection in China: a descriptive study. Lancet Infect Dis. 2015;15(2):196-203.
2. Wen B, Jian R, Zhang Y, Chen R. Simultaneous detection of *Anaplasma marginale* and a new *Ehrlichia* species closely related to *Ehrlichia chaffeensis* by sequence analyses of 16S ribosomal DNA in *Boophilus microplus* ticks from Tibet. J Clin Microbiol. 2002;40(9):3286-90.
3. Parola P, Paddock CD, Socolovschi C, Labruna MB, Mediannikov O, Kernif T, et al. Update on tick-borne rickettsioses around the world: a geographic approach. Clin Microbiol Rev. 2013;26(4):657-702.
4. Platonov AE, Karan LS, Kolyasnikova NM, Makhneva NA, Toporkova MG, Maleev VV, et al. Humans infected with relapsing fever spirochete *Borrelia miyamotoi*, Russia. Emerg Infect Dis. 2011;17(10):1816-23.
5. Chu CY, Jiang BG, Liu W, Zhao QM, Wu XM, Zhang PH, et al. Presence of pathogenic *Borrelia burgdorferi* sensu lato in ticks and rodents in Zhejiang, south-east China. J Med Microbiol. 2008;57(Pt 8):980-5.
